# Temporal dissociation between heart rate recovery and heart rate variability recovery after submaximal exercise in healthy older adults

**DOI:** 10.1186/s11556-026-00432-y

**Published:** 2026-08-10

**Authors:** Gulsah Sahin, Feyza Mutlay

**Affiliations:** 1 Faculty of Sport Sciences, Çanakkale Onsekiz Mart University, Çanakkale, Turkey; 2 Faculty of Medicine, Department of Internal Medicine, Division of Geriatrics, Çanakkale Onsekiz Mart University, Çanakkale, Turkey

**Keywords:** HRR, HRV, Aging, Autonomic function, Exercise recovery

## Abstract

**Background:**

Heart rate recovery (HRR) is commonly used to evaluate post-exercise recovery. However, it might not fully represent the recovery of heart rate variability-derived autonomic indices in older adults. This study examined whether heart rate and heart rate variability-derived indices of cardiac autonomic modulation show divergent recovery patterns following submaximal exercise in healthy older adults.

**Methods:**

Fifty-eight community-dwelling adults aged 60–81 years completed a 5-minute submaximal step test following a 10-minute seated resting period. Heart rate (HR) and heart rate variability (HRV) parameters (RMSSD, pNN50, mean RR, SDNN, LF, HF, LF/HF ratio) were recorded during a 10-minute passive recovery period using a chest strap (Polar H10) monitor and analyzed with Kubios HRV software (version 4.0.3). Recovery dynamics were evaluated by comparing baseline values with the final 5-minute segment of the recovery phase.

**Results:**

Heart rate and mean RR approached pre-exercise resting values during recovery, corresponding to 97.3% and 97.2% recovery, respectively. In contrast, RMSSD and pNN50 showed lower recovery ratios, corresponding to 79.8% and 51.1% of pre-exercise resting values, respectively. ΔHR showed a very strong association with Δ mean RR (r _s_ = -0.932) but only moderate correlations with HRV-derived indices (ΔRMSSD: rs= -0.478; ΔHF: rs = -0.330). Recovery of HRV-derived indices was lower in the older-old group (75–81 years) and was associated with advancing age (ΔRMSSD: rs = -0.312; ΔHF: rs = -0.441).

**Conclusion:**

In healthy older adults, heart rate approached pre-exercise resting values during the post-exercise recovery period, whereas several HRV-derived indices remained incompletely restored. These findings suggest that heart rate and HRV-derived indices may follow different recovery trajectories after submaximal exercise. Because autonomic function was assessed using non-invasive measures, these findings should not be interpreted as direct evidence of a causal dissociation between heart rate recovery and parasympathetic reactivation.

## Introduction

Heart rate recovery (HRR) is widely recognized as an indicator of early parasympathetic reactivation, reflecting the rate at which heart rate declines following exercise [1]. Also, it remains a clinically relevant marker for autonomic dysfunction and exercise intolerance in patients with heart failure and cardiovascular diseases [2]. However, HRR represents only one component of the post-exercise recovery process [3]. The decrease in heart rate after exercise is regulated by several physiological mechanisms, including sympathetic withdrawal, parasympathetic reactivation, removal of metabolic byproducts, and thermoregulatory adjustments [4, 5].

Although heart rate (HR) often declines rapidly after exercise, this early chronotropic recovery does not necessarily indicate complete recovery of cardiac autonomic modulation. The combined effects of parasympathetic reactivation, sympathetic withdrawal, baroreflex adjustments, and residual metabolic or thermoregulatory stress post-exercise heart rate. In contrast, heart rate variability (HRV) indices such as RMSSD and HF power are commonly interpreted as non-invasive markers of cardiac vagal modulation. They may show slower recovery kinetics after exercise. Therefore, a rapid return of heart rate toward resting values may coexist with incomplete recovery of HRV indices. This distinction provides the physiological rationale for examining whether heart rate recovery and HRV recovery follow different post-exercise recovery patterns in older adults [4, 6–8]. Despite this physiological distinction, HRR remains widely used in clinical practice as a reliable indicator of post-exercise recovery [9–11].

HRV provides detailed insight into autonomic recovery by quantifying millisecond fluctuations between successive R-R intervals [12]. In particular, RMSSD is widely recognized as a reliable marker of short-term vagal modulation [13–15]. However, aging is associated with a progressive decline in cardiovagal baroreflex sensitivity and overall autonomic flexibility, which leads to a significant decrease in baseline HRV indices [16–18]. In addition to age-related decreases in HRV, exercise-induced transient autonomic suppression may be further exacerbated following physical exertion. This condition may represent a potential cardiac safety concern, especially in older adults and individuals with impaired cardiovascular adaptability [19, 20].

Therefore, in older adults, decisions regarding resumption of exercise or adjustment of exercise workloads based on HR recovery should be interpreted with caution from a cardiac safety perspective [21]. Despite its potential clinical relevance, a definitive clinical threshold for RMSSD that reflects adequate autonomic recovery following exercise in older adults has not yet been established. Although several studies have investigated the relationship between HRR and parasympathetic modulation, the findings remain inconclusive, largely due to methodological heterogeneity and differences in study populations [8]. Furthermore, the majority of existing research has either evaluated HRR as an independent prognostic marker [3, 10, 22] or focused on HRV recovery in primarily young or athletic populations, typically following maximal or high-intensity exercise protocols [6, 23, 24]. Studies in young healthy adults indicate that heart rate generally declines rapidly during the early post-exercise recovery phase, whereas HRV-derived vagal indices such as RMSSD and HF power may remain suppressed for longer, suggesting that these measures provide complementary information about post- exercise recovery. However, most available evidence comes from young or physically trained individuals, and it is unclear. The interpretability of the available evidence is further limited by heterogeneity in HRV analytical approaches, relatively small sample sizes, and the lack of quantitative models describing recovery kinetics [25–28].

In particular, systematic investigations examining the temporal recovery patterns of heart rate and HRV-derived indices following commonly prescribed submaximal exercise in community-dwelling healthy older adults remain limited [18, 29]. Therefore, clarifying whether heart rate and HRV-derived indices show divergent recovery patterns in older adults is of considerable importance from both physiological and clinical perspectives. We hypothesized that following submaximal exercise, heart rate would approach resting values earlier than HRV-derived indices of cardiac autonomic modulation in healthy older adults. Therefore, this study aimed to examine whether heart rate and HRV-derived indices show divergent recovery patterns during the post-exercise period. Clarifying this distinction may provide useful information for interpreting non-invasive post-exercise recovery monitoring in older adults. It may help guide future studies on recovery assessment after submaximal exercise and exercise prescription in aging populations.

## Method

### Study design

The study was designed as an observational, cross-sectional investigation with a within-subject repeated-measures design. HRV parameters were monitored using a Polar H-10 chest-strap device (Polar Electro, Kempele, Finland) [30, 31]. Data were recorded at a sampling rate of 1000 Hz to ensure sufficient temporal resolution for accurate detection of R-R intervals. R-R intervals data were analyzed using Kubios HRV software (version 4.0.3 Kubios Oy, Kuopio, Finland) [32, 33]. Data was for three phases: the baseline phase (resting), the exercise phase (submaximal test), and the post-exercise phase (recovery). Blood pressure was periodically monitored before the exercise protocol and during the recovery phases using an automated upper-arm electronic BP monitor (Omron M3 Comfort-HEM-7155-E). The physical activity level was assessed with the Physical Activity Scale for the Elderly (PASE) [34, 35]. Sleep quality was evaluated using the Pittsburgh Sleep Quality Index (PSQI) [36, 37]. All measurements were conducted between 08:30 and 11:00 under standardized environmental conditions (22–24 °C). Participants were instructed to refrain from heavy meals and caffeine consumption for at least 3 h before testing and to avoid strenuous physical activity for at least 12 h before the measurements. Participants were also asked to empty their bladders before the test protocol. All measurements were performed in a quiet and controlled environment. The study was conducted in accordance with the principles of the Declaration of Helsinki. The study protocol was approved by the University Health Sciences Institutional Ethics Committee (date: 2026, number: 08/14, project ID: 2026-YONP-0009).

### Participants

Community-dwelling older adults aged 60–81 years were recruited through community advertisements and announcements at the University Healthy Aging Research Center and University Hospital. Each participant underwent clinical screening by a specialist medical doctor. Individuals were excluded if they had a history of cerebrovascular disease with residual deficits, severe musculoskeletal or neurological conditions affecting gait or balance, uncorrected visual impairments, active vestibular disorders, cardiac arrhythmias, cognitive impairments (Montreal Cognitive Assessment score < 26), unstable medical conditions, current smoking habits, or if they were under the influence of sedative medications. Participants using medications known to affect heart rate kinetics or autonomic regulation significantly were also excluded. These included beta-blockers, non-dihydropyridine calcium channel blockers (verapamil or diltiazem), antiarrhythmic agents, ivabradine, centrally acting sympatholytic agents, and anticholinergic medications. Participants taking statins or non-steroidal anti-inflammatory drugs (NSAIDs) were not excluded because their impact on autonomic regulation is generally considered minimal and varies across studies. Individuals with HbA1c levels ≥ 6.5% within the past 3 months were excluded. Medication use and self-reported controlled hypertension were recorded during the screening interview and reported as descriptive clinical characteristics. RR interval recordings with over 10% artifacts were excluded from analysis. A total of 70 individuals were invited to participate, but 12 were excluded for not meeting the eligibility criteria. The final sample included 58 participants (35 women, 23 men). The sample size was determined based on an a priori power analysis.

### Experimental procedure

The HRV monitor chest-strap was positioned over the pectoralis major muscles, with the xiphoid process serving as an anatomical reference point. Measurements were obtained during three different phases:

1. Baseline: Participants were seated comfortably and rested for 10 min. The final 5 min of this period were used as the reference segment for HR and HRV analysis.
2. Submaximal Step Test: Participants performed a 5-minute submaximal stepping test on a 10-cm platform at a cadence of 72 steps.min^− 1^, paced by a digital metronome (auditory pacer). This workload corresponds to approximately 3–4 METs ( ≈ 11.4 ml.kg^− 1^.min^− 1^), according to the ACSM stepping metabolic equation, and was performed as a four-beat stepping cycle (up-up-down-down) at a cadence of 18 step cycles.min^− 1^.

VO_2_ = (0.2 x *f*) + (1.33 × 1.8 x *f* x *H*) + 3.5.

Where f represents stepping frequency and H represents step height. Step tests were selected because they provide a safe and practical method for eliciting cardiovascular responses under submaximal exercise conditions while generating sufficient autonomic stress for physiological assessment [5, 38]. For safety, all tests were conducted under expert supervision, and a fixed support bar was placed adjacent to the platform to assist with balance if needed. Exercise intensity was selected to fall within the moderate-intensity range recommended in previous literature [39–41]. The pilot study (*n* = 10; age: 72.1 ± 3.4 years) was conducted before the main study to confirm that the protocol elicited a submaximal autonomic response in older adults. Heart rate was continuously monitored in real time throughout the protocol. The heart rate value recorded at the final moment of the 5-minute stepping test was extracted as the end-exercise heart rate to contextualize the subsequent heart rate recovery response. Exercise intensity was verified against the predicted maximal heart rate (HR max) calculated using Tanaka formula [208−(0.7×age)] [42].

During the stepping test and immediately after exercise, participants’ perceived exertion was verbally monitored as part of real-time safety and intensity supervision. However, perceived exertion values were not systematically recorded or retained for statistical analysis.

For age-based subgroup analyses, participants were categorized into two groups: younger-old adults aged 60–74 years and older-old adults aged 75–81 years. This categorization was defined prior to subgroup analyses to examine whether post-exercise heart rate and HRV recovery responses differed by age group.

The pilot study confirmed that the protocol elicited an average response of 65.2% of predicted HR max and a perceived exertion of 13.1 ± 0.7 on the Borg Rating of Perceived Exertion (RPE) scale, indicating a “somewhat hard” effort. Although individual VO_2_max was not measured, these results demonstrate that the protocol met the criteria for submaximal exercise. These findings indicate that the test protocol is consistent with moderate-intensity exercise guidelines for older adults and provides sufficient physiological stress to elicit measurable autonomic responses [43].

Step height was selected because it provides moderate-intensity exercise without imposing excessive lower-limb load, particularly in older adults and individuals with low fitness levels [44, 45].

III. Recovery Phase: Immediately after the step test, participants underwent passive recovery in a seated position for 10 min, during which heart rate and HRV were continuously recorded. Participants were instructed to remain seated, avoid speaking, and breathe spontaneously throughout the recovery period. Due to the non-stationary dynamics in R-R intervals associated with the rapid initial decline in heart rate, the first 5 min of post-exercise data were excluded from HRV analyses. The final 5-minute segment, spanning minutes 5–10 post-exercise, was used for HRV evaluation [12, 46, 47]. Depending on the analysis method, heart rate data from the first 60–120 s post-exercise are typically used to calculate HRR. In contrast, different time windows have been employed in the literature for post-exercise HRV assessment [4, 26, 48]. A 10-minute recovery period was selected to capture both the early chronotropic recovery phase and a later 5 -minute short-term HRV analysis window, while maintaining feasibility and participant comfort in older adults and minimizing potential confounding effects of prolonged immobility, such as psychological discomfort or restlessness.

### HR and HRV measurements- preprocessing and parameters

To avoid potential effects of the Polar software’s autonomic filtering on HRV indices, raw R-R interval data were exported and analyzed using Kubios HRV software. Before analysis, the data were visually inspected to ensure: (a)the absence of prominent RR outliers, (b)physiological consistency between successive RR intervals, (c)stable mean values, (d)unimodal distribution, and (e)preservation of physiological variability. Artifact correction was performed using Kubios HRV’s threshold-based correction algorithm. To preserve the inherent physiological variability of cardiovascular dynamics in older adults and to avoid artificial smoothing of autonomic signals (over-smoothing), a low-level correction was applied [27, 28]. R-R series were also processed using the Smoothness Priors method (λ = 500), a commonly applied setting for rapid HRV analyses [49]. For HRV analysis, the following time-domain parameters were calculated: HR, RMSSD, SDNN, pNN50, and Mean RR. Frequency-domain analysis included LF, HF, and the LF/HF ratio [12]. Frequency-domain analysis was performed using the default Fast Fourier Transform (FFT)-based spectral analysis settings in Kubios HRV software. Low-frequency power, high-frequency power, and the low-frequency/high-frequency ratio was calculated as frequency-domain indices.

The physiological interpretation of the HRV variables was considered as follows. Mean RR reflects the average interval between consecutive normal heartbeats and is inversely related to heart rate. SDNN represents overall short-term HRV, whereas RMSSD and pNN50 are commonly interpreted as time-domain indicators of cardiac vagal modulation [12]. In the frequency domain, HF power is generally considered to reflect respiratory-related vagal modulation, whereas LF power may reflect a combination of sympathetic, parasympathetic, and baroreflex-related influences [7, 12, 21]. The LF/HF ratio was interpreted cautiously as an index of sympathovagal balance, recognizing that this measure is influenced by respiratory behavior and hemodynamic regulation [7, 12, 21].

LF and HF power were reported in absolute units rather than normalized units because the study focused on pre- to post-exercise changes in spectral power magnitude of spectral power within each frequency band. Normalized units can emphasize the relative distribution of LF and HF power but may obscure changes in total spectral power and are sensitive to changes in the denominator. Therefore, absolute LF and HF values were retained, and the LF/HF ratio was interpreted cautiously as a descriptive frequency-domain index rather than as a direct measure of sympathovagal balance.

Time-domain indices were used because they provide simple, clinically interpretable, and widely accepted measures of post-exercise recovery and short-term HRV. In this study, early heart rate recovery indices reflected the initial chronotropic response after exercise, whereas RMSSD, SDNN, and pNN50 calculated from the final 5 min of recovery provided a more stable short-term HRV segment. However, this approach also has limitations. Early HRR indices do not isolate the specific autonomic mechanisms underlying heart rate decline, and 5-minute HRV segments may not fully capture the dynamic beat-to-beat recovery process immediately after exercise. Therefore, these indices were interpreted as non-invasive recovery markers rather than direct measures of parasympathetic or sympathetic activity.

## Respiratory rate control and spectral analysis sensitivity

The controlled breathing approach was not chosen to avoid creating an artificial physiological state that could mask the body’s natural post-exercise responses and neuromuscular control [50]. To ensure the physiological validity of HRV spectral components, respiratory rate was monitored during each analysis period using the ECG-derived respiration (EDR) algorithm implemented by Kubios software. This algorithm calculates respiratory frequency by analyzing R-R interval fluctuations associated with respiratory sinus arrhythmia (RSA) [32]. Previous studies have shown that EDR provides meaningful respiration-related information from the ECG signal and that components are consistent with measured respiratory activity in terms of spectral content [51–53]. This control is particularly critical for the accuracy of the high-frequency (HF) power spectrum (0.15–0.40), which is highly sensitive to breathing rate.

Respiratory frequency was not experimentally paced during the protocol. ECG-derived respiration estimates obtained with Kubios were used only as a general monitoring indicator, not as a formal exclusion criterion for spectral analysis. Therefore, no participants were excluded from the spectral analysis of respiratory frequency. Because direct respiratory flow or tidal volume was not measured, the possibility that respiratory behavior influenced frequency-domain HRV indices including potential overlap with the LF band, cannot be completely ruled out.

### Statistical analyses

Statistical analyses were performed using SPSS v26.0 (IBM Corp., Armonk, NY, USA). The sample size was determined via a priori power analysis using G*Power (v3.1.9.7) based on an effect size d = 0.38, α = 0.05, and (1-β) = 0.80, resulting in *N* = 58. The normality of variables was assessed using the Shapiro-Wilk test. Descriptive statistics were reported as mean, standard deviation (±), and median (interquartile range, IQR). The difference between pre-exercise and post-exercise parameters was analyzed using the Wilcoxon Signed-Rank Test. Between-group comparisons (age-gender) were analyzed with the Mann-Whitney U test. The relationship between variables was evaluated using Spearman’s Rank correlation coefficient test. Categorical variables, including sex distribution, controlled hypertension, and regular medication use, were compared between age groups using Fisher’s exact test. The significance was set at *p* < .05. To quantify overall post-exercise autonomic changes and individual variability, delta (Δ) values were calculated as:

Δ = Post-exercise value- Pre-exercise resting value.

Relative recovery was assessed using Recovery Ratio and Recovery Gap metrics. The Recovery Ratio (%) was calculated as:

Recovery Ratio (%) = (Post-exercise recovery value/ pre-exercise resting value) x 100.

The Recovery Gap (%) was defined as the percentage difference between the pre-exercise resting value and the post-exercise recovery value. It was calculated as: Recovery Gap (%) = [(Pre-exercise resting value -post-exercise recovery value) / pre-exercise resting value] x 100. A positive Recovery Gap value indicates that the post-exercise value remained below the pre-exercise resting value. In contrast, a value closer to zero indicates more complete recovery toward the resting value. Negative values indicate that the post-exercise value exceeded the pre-exercise resting value. To formally examine the descriptive discrepancy between heart rate recovery and HRV-derived vagal recovery indices, paired comparisons were performed between heart rate recovery percentage and the recovery percentages of RMSSD, pNN50, and HF power. The Wilcoxon signed-rank tests were used. The resulting p values were adjusted using the Benjamini-Hochberg false discovery rate procedure.

Physical activity levels (PASE) and sleep quality (PSQI) were reported descriptively. Differences between groups in these parameters were assessed using independent t-tests. Effect sizes for Mann-Whitney U tests were calculated as:

r = Z /√ N.

Where Z is the standardized test statistic, and N is the total number of participants. Effect sizes were classified by Cohen (1988) as small (0.10–0.29), medium (0.30–0.49), and large (≥0.50).

## Results

Demographic characteristics, physical activity levels, and sleep quality scores of all participants are presented by age group in Table 1. No significant differences were observed between the 60–74 years group (*n* = 39) and the 75–81 years group (*n* = 19) in height (*p* = .060), body weight (*p* = .455), and body mass index (*p* = .169). Similarly, physical activity levels (PASE, *p* = .710) and overall sleep quality (PSQI, *p* = .829) were comparable between the two groups.

**Table 1:** General Characteristics of Participants

Variable	Total (*n* = 58)	60–74 Years (*n* = 39)	75–81 Years (*n* = 19)	*p*
Age (years)	71.21 ± 6.2	67.20 ± 3.9	78.21 ± 2.2	0.001
Height (cm)	163.43 ± 11.1	160.5 ± 9.6	166.1 ± 12.1	0.060
Weight (kg)	68.74 ± 10.4	67.7 ± 9.7	69.9 ± 11.2	0.455
BMI (kg.m^− 2^)	25.81 ± 3.01	26.2 ± 3.11	25.1 ± 1.82	0.169
PASE Score	135.2 ± 18.4	136.7 ± 19.5	134.7 ± 15.9	0.710
PSQI Score	5.02 ± 1.4	5.3 ± 1.5	5.4 ± 1.3	0.829
Sex, female/ male, n (%)	35/23 (60.3/39.7)	28/11 (71.8/28.2)	7/12 (36.8/63.2)	0.021
Controlled hypertension, n (%)	15 (25.9)	9 (23.1)	6 (31.6)	0.533
Regular medication, n (%)	23(39.7	12(30.8)	11(57.9)	0.085

Values are presented as mean ± SD or n (%), as appropriate. *PSQI* Pittsburgh Sleep Quality Index, *BMI* Body Mass Index, *PASE* Physical Activity Scale for the Elderly

At the pre-exercise resting assessment, heart rate and most HRV parameters did not differ significantly between the younger-old and older-old groups, including HR, mean RR, RMSSD, SDNN, HF power, and the LF/HF ratio (all *p* > .05). pNN50 was close to the conventional threshold for statistical significance (*p* = .050), whereas LF power did not differ significantly between groups (*p* = .062) Table 2.

**Table 2:** Pre-exercise resting and post-exercise recovery values for heart rate and heart rate variability parameters in all participants

Parameters *N* = 58	Pre-exercise Mean ± Median (IQR)		Post-exercise Mean ± Median (IQR)		*p*	z	Effect Size (*r*)
HR (beat.min^− 1^)	68.55 ± 7.34	66.55 (63.5–73.3)	70.65 ± 8.08	68.34 (65.6–77.2)	< 0.001*	-4.95	0.65
RMSSD (ms)	19.88 ± 18.60	15.04 (12.1–21.2)	16.97 ± 16.39	12.00 (9.3–18.6)	< 0.001*	-4.93	0.65
Mean RR (ms)	885.10 ± 90.39	902.12 (817.6-948.6)	857.80 ± 94.48	877.17 (774.3-917.7)	< 0.001*	-5.34	0.70
SDNN (ms)	18.45 ± 10.96	15.18 (13.0-19.9)	17.42 ± 10.34	14.88 (11.5–20.3)	0.106	-1.61	0.21
pNN50 (%)	3.37 ± 10.30	0.45 (0.1–1.4)	3.47 ± 11.48	0.23 (0.0-1.2)	0.027*	-2.22	0.29
LF (ms²)	130.31 ± 140.68	86.29 (51.7-153.1)	168.46 ± 251.22	96.87 (67.4-174.9)	0.056	-1.91	0.25
HF (ms²)	183.96 ± 527.70	66.64 (51.0-164.1)	147.18 ± 464.65	60.88 (28.2-104.8)	0.002*	-3.16	0.42
LF/HF Ratio	1.44 ± 0.99	1.05 (0.7–2.1)	2.25 ± 1.54	2.13 (1.2–2.7)	< 0.001*	-3.63	0.48

IQR: interquartile range (25–75 percentiles), *p < .05

Comparison of pre-exercise and post-exercise autonomic parameters in all participants (*n* = 58) revealed significant differences in HR, mean RR, RMSSD, pNN50, HF, and the LF/HF ratio (*p*< .05). During the 5-minute post-exercise segment, RMSSD (*p* < .001, *r* = .65) and HF (*p* = .002, *r* = .42) remained significantly lower than pre-exercise values. For the FL/HF ratio, there was a significant difference compared to the pre-exercise value (*p*<.001, *r*=.48). No significant differences were observed for SDNN (*p* = .106) or LF (*p* = .056) relative to pre-exercise (*p*>.05).

### Relationship Between HR and HRV Parameters

**Fig. 1:**
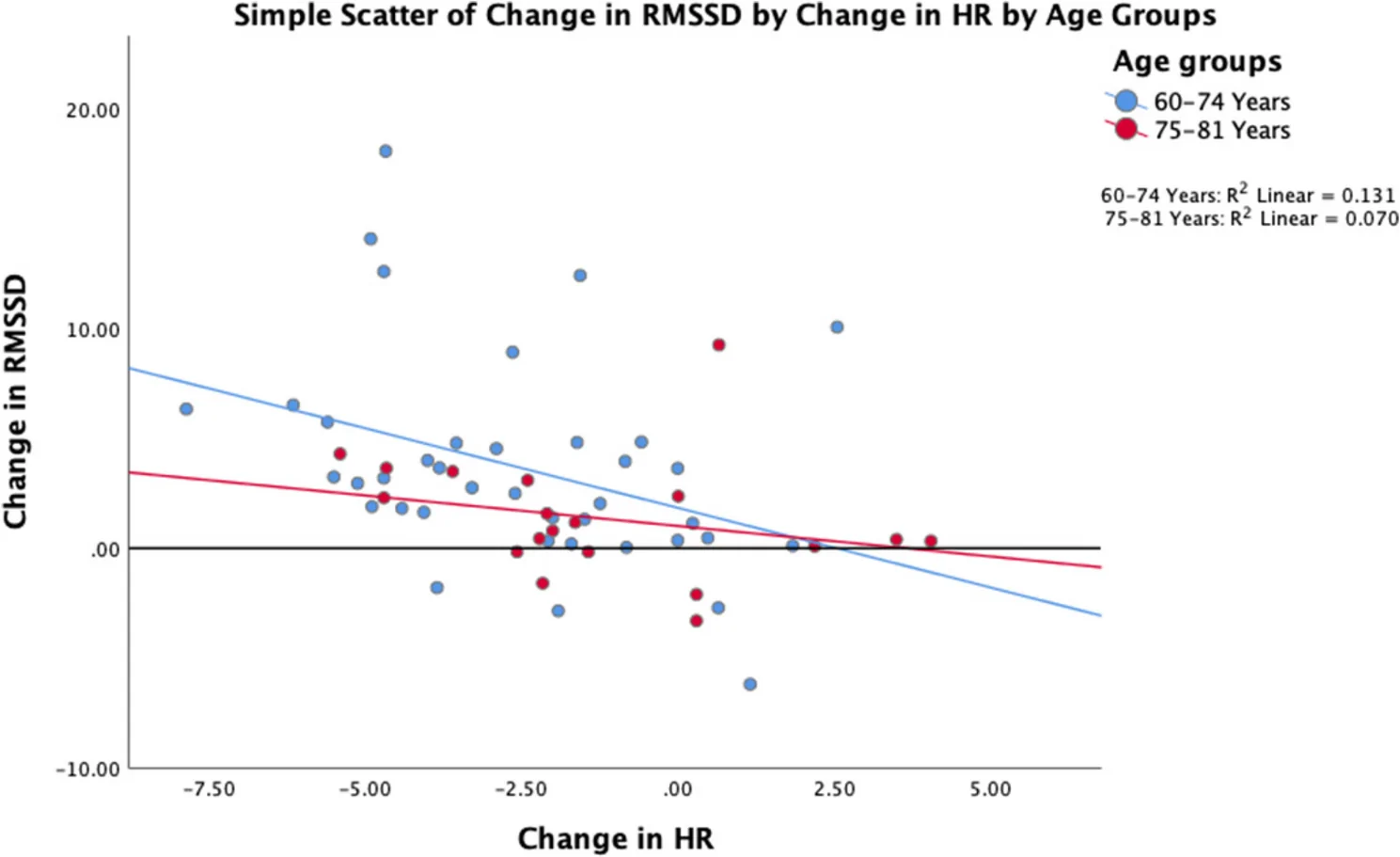
Changes in post-exercise Δ RMSSD and Δ HR relative to pre-exercise across age groups

The relationship between heart rate recovery (ΔHR) and autonomic recovery parameters was assessed using Spearman’s rank correlation analysis (r _s_). A significant moderate negative correlation was observed between ΔHR and ΔRMSSD (*r*_s_ = -0.478, *p* < .001). ΔHR was strongly correlated with Δ mean RR (*r*_s_ = -0.932), and moderately correlated with ΔpNN50 (*r*_s_ = -0.283) and ΔHF (*r*_s_ = -0.330). A moderate positive correlation was found between ΔLF/HF ratio and ΔHR (*r*_s_ = 0.393, *p* = .002). No significant association was observed between ΔLF and ΔHR (*p* = .761) Table 3 and Fig. 1.

**Table 3:** Spearman Correlation Coefficients Between Heart Rate Recovery (ΔHR) and Heart Rate Variability Parameters (Δ) (*n* = 58)

Autonomic Parameters (Δ)	Correlation with Δ HR (*r*_s_)	*p*-value
Δ RMSSD	-0.478	< 0.001**
Δ mean RR	-0.932	< 0.001**
Δ SDNN	-0.276	0.036*
Δ pNN50	-0.283	0.031*
Δ LF	-0.041	0.761
Δ HF	-0.330	0.011*
Δ LF/HF Ratio	0.393	0.002**

r _s_: Spearman’s rho. Δ (Delta) represents the change from pre-exercise to Post-exercise recovery * *p* < .05; ** *p* < .01

### Acute percent (%) change analysis for post-exercise parameters

Acute percent changes and recovery relative to the pre-exercise resting values were analyzed. Heart rate (HR) and mean RR approached pre-exercise values, corresponding to 97.3% and 97.2%, respectively. In contrast, RMSSD and pNN50 showed lower recovery ratios, corresponding to 79.8% and 51.1% of pre-exercise resting values, respectively. The LF/HF ratio increased to 102.3%, HF power decreased by 8.64%, and LF power increased by 12.2%. Formal paired comparisons of recovery percentages showed that the HR recovery percentage was significantly higher than the RMSSD recovery percentage (z=-5.153, p_FDR < 0.001) and the HF recovery percentage (z=-2.807, p_FDR=0.008). The difference between the HR recovery percentage and the pNN50 recovery percentage did not reach statistical significance after FDR correction (z=-1.868, p_FDR=0.062).

At the pre-exercise resting assessment, heart rate and most HRV parameters did not differ significantly between the younger-old and older-old groups, including HR, mean RR, RMSSD, SDNN, HF power, and LF/HF ratio (all *p*>.05). pNN50 showed a borderline between-group difference (*p*= .050), while LF power showed a non-significant trend (*p*= .062).

Age-group comparisons of post-exercise recovery responses are presented in Table 4.

**Table 4:** The Differences and relationship between heart rate variability parameters across age groups

Variables	60–74 Years (*n* = 39) Median (IQR)	75–81 years (*n* = 19) Median (IQR)	Z	*P*	Effect size (*r*)	Correlation with age (*r*_s_)	Correlation (*p*)
Δ HR (bpm)	-2.60 (-5.5–1.2)	-2.00 (-4.2–0.8)	-1.52	0.127	0.20	0.166	0.214
Δ RMSSD (ms)	2.96 (1.4–4.2)	0.80 (0.2–1.9)	-2.18	0.029*	0.28	− 0.312*	0.017
Δ mean RR (ms)	31.82 (15.5–55.0)	22.42 (10.2–38.4)	-1.30	0.193	0.17	− 0.148	0.266
Δ SDNN (ms)	1.50(-1.2-3.2)	0.39 (-1.8-1.8)	-1.33	0.182	0.17	− 0.203	0.126
Δ pNN50 (%)	0.10 (0.0-0.6)	0.10 (-1.5-0.4)	− 0.44	0.660	0.05	− 0.174	0.192
Δ LF (ms²)	-18.60 (-48.2-22.0)	-5.00 (-15.5-10.5)	− 0.62	0.534	0.08	0.109	0.415
Δ HF (ms²)	28.00 (12.5–45.0)	-3.90 (-12.0–5.5)	-2.74	0.006**	0.36	− 0.441**	0.001**
Δ LF/HF Ratio	-1.35 (-2.8–0.4)	-0.73 (-1.5–0.1)	-1.53	0.125	0.20	0.273*	0.038*

Data are presented as Median (Interquartile Range: 25th -75th percentiles). *p <.05, **p <.01

Participants were categorized by age groups: 60–74 years (younger-old) and 75–81 years (older-old). Parasympathetic (vagal) reactivation in the older-old group (75–81 years) was significantly lower than in the younger-old group (ΔRMSSD: *p* = .029, *r* = .28; ΔHF: *p* = .006, *r* = .36). Notably, the ΔHF parameter in the older-old group had a negative median value (Md = -3.90), whereas the younger-old group showed a positive recovery trend (Md = 28). ΔHR did not differ significantly between groups (*p* = .127, *r* = .20). The association between age and autonomic dissociation during post-exercise recovery was further examined using Spearman’s rank correlation (Table 4). Age was significantly negatively correlated with ΔRMSSD (r _s_ = -0.312, *p* = .017) and ΔHF (r _s_ = -0.441, *p* = .001). No significant associations were found between age and ΔHR (*p* = .214) or Δ mean RR (*p* = .266). A positive correlation was observed between age and ΔLF/HF ratio (r _s_ = 0.273, *p* = .038).

Post-exercise recovery dynamics were compared between female and male groups (female, *n* = 35; male, *n* = 23). No statistically significant differences were observed for any measured parameters (all *p* > .05). Both ΔHR (*p* = .930) and ΔRMSSD (*p* = .382), as well as ΔHF (*p* = .169), showed similar trends in female and male participants.

## Discussion

The present findings indicate that, during post-exercise recovery, heart rate approached pre-exercise values, whereas several HRV-derived indices of cardiac autonomic modulation remained incompletely restored. Specifically, RMSSD and HF power remained lower than resting values, while the LF/HF ratio increased during the recovery period. A second important finding was that recovery of HRV-derived indices varied by age group. Specifically, the older-old group showed less complete recovery in RMSSD-and HF-derived recovery responses compared with the younger-old group, suggesting that advanced age may be associated with less complete restoration of non-invasive autonomic recovery markers after submaximal exercise. These findings suggest that heart rate recovery may not fully capture the recovery pattern of HRV-derived autonomic markers after submaximal exercise in healthy older adults [4, 7, 21]. However, because HRV-derived indices are indirect measures and may be influenced by respiratory behavior, baroreflex dynamics, and hemodynamic adjustments, these results should be interpreted cautiously [4, 7].

Although previous pharmacological studies have demonstrated that early heart rate recovery is predominantly mediated by vagal reactivation, with sympathetic withdrawal playing a secondary role [54], the present study was not designed to isolate the underlying autonomic mechanisms. Rather, our findings suggest that heart rate normalization may occur earlier than the recovery of HRV- derived indices of cardiac autonomic modulation following submaximal exercise in healthy older adults. Therefore, the observed divergence should be interpreted as a non-invasive recovery pattern rather than direct evidence of delayed parasympathetic reactivation or causal autonomic dissociation.

The main finding of this study is that heart rate and HRV-derived autonomic indices showed different apparent recovery patterns following submaximal exercise. During the recovery period, heart rate and mean RR approached pre-exercise resting values, reaching approximately 97% of their pre-exercise level. In contrast, RMSSD and pNN50 remained less completely restored, corresponding to 79.8% and 51.1% of pre-exercise resting values, respectively. Formal paired comparisons further showed that heart rate recovery percentage was significantly higher than the RMSSD and HF recovery percentage after FDR correction. In contrast, the difference between heart rate recovery percentage and pNN50 recovery percentage did not reach statistical significance. This pattern suggests that the normalization of heart rate may not fully reflect the recovery of selected HRV-derived markers of cardiac autonomic modulation in healthy older adults [7, 12, 21, 24]. The increase in the LF/HF ratio during recovery may indicate a shift in HRV-derived sympathovagal balance; however, this interpretation should be approached with caution because frequency-domain indices are influenced by respiratory behavior, baroreflex dynamics, and other hemodynamic factors [12, 21]. Therefore, the observed discrepancy between heart rate and HRV-derived indices should be interpreted as a divergent non-invasive recovery pattern rather than as direct evidence of persistent sympathetic dominance, delayed parasympathetic reactivation, or increased acute cardiac risk [7, 21, 24].

The physiological mechanisms underlying the different recovery trajectories of heart rate and HRV-derived indices cannot be directly determined from the present study. Previous pharmacological evidence indicates that early heart rate recovery is strongly influenced by vagal reactivation, while sympathetic withdrawal and baroreflex-related adjustments may also contribute to the broader recovery process [54]. In the present study, correlation analyses further show that ΔHR was very strongly associated with Δ mean RR (r _s_ = -0.932), whereas its associations with HRV-derived indices were only moderate, including ΔRMSSD (r _s_ = -0.478) and ΔHF (r _s_ = -0.330). Similarly, the positive association between ΔHR and ΔLF/HF ratio (r _s_ = 0.393) may suggest a shift in HRV-derived sympathovagal balance during recovery. However, these associations should be interpreted cautiously, because HRV-derived indices are influenced by respiratory behavior, baroreflex regulation, hemodynamic adjustments, and residual metabolic or thermal stress [7, 21, 55]. Therefore, the weaker associations between ΔHR and ΔRMSSD or ΔHF should be interpreted as evidence that heart rate and HRV-derived indices do not fully overlap as non-invasive recovery markers, rather than as proof of distinct autonomic mechanisms [55].

Another key finding of this study was that of HRV-derived autonomic indices differed across age groups (Table 4). Specifically, the older older-old group (75–81 years) showed less complete recovery in ΔRMSSD and ΔHF than the younger-old group (60–74 years). In addition, age was negatively correlated with ΔRMSSD and ΔHF, suggesting that advancing age may be associated with reduced recovery of HRV-derived autonomic markers after submaximal exercise [20, 42, 56, 57]. However, this interpretation should be approached with caution as pre-existing age-related differences in resting autonomic modulation may influence post-exercise recovery responses. The comparable physical activity and sleep quality scores between age groups suggest that lifestyle-related differences may not fully explain this pattern, although the absence of a younger adult comparison group limits age-specific causal interpretation [58].

Evidence from young healthy populations indicates that heart rate and HRV may show different recovery characteristics after submaximal exercise. Barak et al., (2020) examined young healthy men who underwent a 5-minute submaximal cycling protocol. They assessed HRV using 5-minute R-R interval segments obtained during the final 5 min of a 10-minute recovery period in different body positions [59]. This study suggests that post-exercise HRV responses in young individuals may be influenced by recovery posture. In addition, Barak et al., (2011) reported that heart rate recovery after submaximal exercise differed according to recovery protocol and training status in young male athletes and non-athletes [60]. However, direct comparison with the present findings should be made cautiously because the present study included older adults, used a submaximal stepping protocol, and assessed HRV during the final 5-minutes of a 10-minute seated recovery period. Therefore, the incomplete restoration of RMSSD and HF power observed in the present study may reflect both the slower recovery kinetics of HRV-derived indices after exercise and age-related reductions in autonomic flexibility.In our study, no statistically significant differences in post-exercise recovery dynamics were detected between female and male participants (all *p* > .05). Similarly, key parameters, including ΔHR (*p* = .930), ΔRMSSD (*p* = .382), and ΔHF (*p*=.169), did not differ significantly between women and men. However, this finding should be interpreted cautiously because the study was not powered to examine sex-specific recovery responses. Therefore, the absence of significant sex differences should not be interpreted as evidence of equivalent recovery patterns between women and men. Future studies with larger, sex-balanced samples are needed to clarify whether sex modifies heart rate and HRV-derived recovery responses in older adults [57].

These findings have implications for post-exercise monitoring and exercise prescription in older adults. In many exercise programs for older adults, recovery duration is often guided primarily by heart rate recovery. However, the present findings suggest that heart rate recovery alone may not fully reflect the recovery pattern of HRV-derived autonomic markers after submaximal exercise. Therefore, HRV-derived indices may provide additional information for non-invasive post-exercise monitoring and recovery assessment [24, 26]. In particular, the delayed recovery of HRV-derived indices observed in older adults suggests that recovery responses may need to be interpreted using multiple physiological markers rather than heart rate alone [18, 61]. Nevertheless, further studies incorporating in measurement of autonomic, hemodynamic, and clinical outcome measures are needed before specific clinical recovery recommendations can be made.

The interpretation of these findings should also consider the characteristics of the study sample. The participants were healthy, community-dwelling healthy older adults, and the findings should not be directly extrapolated to highly physically active or trained older adults, who may exhibit different HRV recovery responses after exercise.

### Limitations

Several limitations should be considered when interpreting the findings of this study. A major limitation of this study is the absence of pharmacological autonomic blockade. Therefore, parasympathetic reactivation and sympathetic withdrawal could not be directly isolated as independent contributors to post-exercise recovery. Although HRV-derived indices such as RMSSD and HF power are commonly used as non-invasive markers of cardiac vagal modulation, they remain indirect measures. They may be influenced by respiration, baroreflex dynamics, hemodynamic adjustments, and concurrent sympathetic modulation. Thus, the observed divergence between heart rate recovery and HRV-derived indices should be interpreted as a non-invasive recovery pattern rather than direct evidence of causal autonomic dissociation. Secondly, autonomic recovery was assessed using RMSSD as an indicator of vagal activity. While RMSSD is a well-known indicator of parasympathetic modulation, it does not directly measure global autonomic balance. Direct physiological measurements, such as circulating catecholamines, were not obtained. Inferences regarding delayed parasympathetic reactivation should be evaluated indirectly. Thirdly, R-R intervals were recorded using a Polar H10 chest band. Although this device has proven to correlate highly with clinical ECG systems [30, 62, 63], its single-channel recording may be more susceptible to artifacts. However, rigorous preprocessing using Kubios HRV software minimized this risk. Finally, respiratory rate was monitored via the EDR algorithm in Kubios [64], but respiratory depth was not directly measured. Individual differences in respiratory mechanics can subtly affect vagal modulation, which should be considered.

## Conclusion

In healthy older adults, heart rate approached resting values during the post-exercise recovery period, whereas several HRV-derived indices of cardiac autonomic modulation remained incompletely restored. In addition, recovery of HRV-derived indices was lower in the older -old age group, suggesting that advanced age may be associated with less complete recovery of non-invasive autonomic markers after submaximal exercise. However, because pharmacological autonomic blockade was not used, these findings should be interpreted as evidence of divergent non-invasive recovery patterns rather than direct proof of delayed parasympathetic reactivation or causal autonomic dissociation. Future studies incorporating pharmacological blockade, controlled respiratory assessment, continuous hemodynamic monitoring, and younger comparison groups are needed to clarify the underlying autonomic mechanisms and the age-specific nature of these recovery patterns. Therefore, these conclusions should be interpreted within the context of community-dwelling healthy older adults and should not be generalized to highly physically active or trained older adults.

## Data Availability

The datasets generated during and/or analyzed during the current study are available from the corresponding author on reasonable request.

## Abbreviations

ACSM: American College of Sports Medicine
BMI: Body mass Index
ECG: Electrocardiogram
EDR: ECG-Derived Respiration
HF: High Frequency
HR: Heart Rate
HRR: Heart Rate Recovery
HRV: Heart Rate Variability
LF: Low Frequency
Mean RR: Mean of R-R intervals
MET: Metabolic Equivalent of Task
NSAIDs: Non-steroidal anti-inflammatory drugs
PASE: Physical Activity Levels for the Elderly
pNN50: The proportion of adjacent NN intervals differing by more than 50 ms
PSQI: Pittsburgh Sleep Quality Index
RMSSD: Root Mean Square of Successive Differences
RPE: Rating of Perceived Exertion
RSA: Respiratory sinus Arrhythmia
SDNN: Standard Deviation of NN intervals
